# A Review on Nanopesticides for Plant Protection Synthesized Using the Supramolecular Chemistry of Layered Hydroxide Hosts

**DOI:** 10.3390/biology10111077

**Published:** 2021-10-21

**Authors:** Syazwan Afif Mohd Zobir, Asgar Ali, Fariz Adzmi, Mohd Roslan Sulaiman, Khairulmazmi Ahmad

**Affiliations:** 1 Department of Plant Protection, Faculty of Agriculture, Universiti Putra Malaysia, Serdang 43400 UPM, Selangor, Malaysia; 2 Institute of Plantation Studies, Universiti Putra Malaysia, Serdang 43400 UPM, Selangor, Malaysia; farizadzmi@upm.edu.my; 3 Centre of Excellence for Postharvest Biotechnology (CEPB), School of Biosciences, University of Nottingham Malaysia, Jalan Broga, Semenyih 43500, Selangor, Malaysia; Asgar.Ali@nottingham.edu.my; 4 Department of Science and Biomedicine, Faculty of Medicine and Health Sciences, Universiti Putra Malaysia, Serdang 43400 UPM, Selangor, Malaysia; mrs@upm.edu.my

**Keywords:** supramolecular chemistry, host–guest, nanopesticides, layered hydroxides, layered double hydroxide

## Abstract

**Simple Summary:**

Nanoscience and nanotechnology offer a new life for conventional pesticides with superior qualities by virtue of the physicochemical properties of the nanosized materials. These properties will improve bioavailability through different kinetics, mechanisms and pathways on their target organisms; enabling them to properly bypass biological and other unwanted resistances and therefore increase plant disease control efficacy. Inorganic two-dimensional (2D) layered hydroxides and layered double hydroxides were used as hosts, to act as functional nanocarriers for the delivery of various pesticides in combating various pests and diseases, in order to aid plant protection. This leads toward a new generation of more effective agrochemicals which are safe to life, humans and the environment.

**Abstract:**

The rapid growth in the human population has triggered increased demand for food supply, and in turn has prompted a higher amount of agrochemical usage to meet the gaps between food production and consumption. The problem with conventional agro-nanochemicals is the reduced effectiveness of the active ingredient in reaching the target, along with leaching, evaporation, etc., which ultimately affect the environment and life, including humans. Fortunately, nanotechnology platforms offer a new life for conventional pesticides, which improves bioavailability through different kinetics, mechanisms and pathways on their target organisms, thus enabling them to suitably bypass biological and other unwanted resistances and therefore increase their efficacy. This review is intended to serve the scientific community for research, development and innovation (RDI) purposes, by providing an overview on the current status of the host–guest supramolecular chemistry of nanopesticides, focusing on only the two-dimensional (2D), brucite-like inorganic layered hydroxides, layered hydroxide salts and layered double hydroxides as the functional nanocarriers or as the hosts in smart nanodelivery systems of pesticides for plant protection. Zinc layered hydroxides and zinc/aluminum-layered double hydroxides were found to be the most popular choices of hosts, presumably due to their relative ease to prepare and cheap cost. Other hosts including Mg/Al-, Co/Cr-, Mg/Fe-, Mg/Al/Fe-, Zn/Cr- and Zn/Cu-LDHs were also used. This review also covers various pesticides which were used as the guest active agents using supramolecular host–guest chemistry to combat various pests for plant protection. This looks towards a new generation of agrochemicals, “agro-nanochemicals”, which are more effective, and friendly to life, humans and the environment.

## 1. Introduction

Agrochemicals are multi-billion-dollar businesses for both the upstream and downstream agroindustries [1], not only providing significant employment to the population but also providing important solutions in food security and other agro-related industries. Unfortunately, attached to these industries are long- and short-term unwanted consequences, such as toxicities to nontarget crops and the users, life and environmental threats due to leaching and uncontrolled applications of the agrochemicals [2,3,4].

The demand for agrochemicals grows year by year due to the direct increase of food demand, which is in turn due to the high population, or due to indirect factors such as climate change, pesticide resistance, etc. This can be overcome by producing a new generation of safer agrochemicals which are life, user and environmentally friendly, by understanding their mechanism of actions, dose–cytotoxicity relationship, translocation mechanisms and omics behavior so that the nature of soil–plant–nanoparticle interactions are fully understood [5].

Nanoscience and nanotechnology based on different disciplines and combined with precision farming can offer a holistic solution for more effective and precise tools for diagnosis, treatment and pest-management control in the modern agricultural sector [6]. The understanding of nanoscience and nanotechnology enables us to increase agrochemical efficacy and reduce the amount used, reduce the frequency of application, minimize nutrient or active-ingredient losses and also improve plant tolerance to various biotic stresses such as drought, heat, and salt [7], and hence optimize the input–output balance of the agricultural products.

For crop protection and management, nanomaterials can be used as efficient delivery agents for various pesticides; insecticides, herbicides, fungicides, defoliant, biocides, etc., and can help us move towards economically viable and ecofriendly agrochemicals [8]. With their controlled release and targeted delivery, this new generation of pesticides can improve the bioavailability of the active ingredients and therefore increase their efficacy compared with their counterparts [9]. Furthermore, in the long run, the updated regulations and safety of current pesticides and their nanoproducts are necessary to protect humankind and the environment.

Over the years, the advantages of nanotechnology applications in agriculture, especially for pesticides, have been uncovered, as shown by the growing trend in research, development and innovation (RDI). Therefore, this review will discuss the use of host–guest supramolecular chemistry for plant disease protection. Due to the intense development that has been made in this area of RDI, we only focus our review on the layered hydroxides and layered double hydroxides as the hosts and various types of pesticides as the guests or active ingredients (AI). In addition, the synthesis methods, kinetics and mathematical models and characterization techniques together with their controlled-release properties are briefly discussed. Moreover, a discussion on the RDI of nanopesticides, especially considering their conventional active ingredients, will also be included in this review.

To the best of our knowledge, no specific review focusing on host–guest supramolecular chemistry using various layered hydroxides as the hosts is available in the open literature. Therefore, it is hoped that this review will fill the gap and serve to update the current advancements in this area of RDI, where nanoscience and nanotechnology coupled with green technology [10] are used for holistic pest management, especially for plant protection.

This review aims to compile and update the current trends and advancements of the next generation of agrochemicals, in particular, the agro-nanofungicides. The manuscript covers the definitions, chemical structures, applications, their synthesis routes and physicochemical characterizations. In addition, some basic principles for their formation, and finally the advancement of the current RDI of these types of agro-nanofungicides for plant protection are also included.

This review also aims to give a general overview of the use of host–guest supramolecular chemistry for the synthesis of fungicide nanodelivery systems, nanopesticides, their advantages and limitations. Due to too many hosts that can be possibly used for this purpose, we confine our review to only the layered hydroxides and layered double hydroxides as the hosts, and various types of fungicide active ingredients as the guests. The following keywords were used in this search: layered hydroxides and/or layered double hydroxides together with either pesticides, antimicrobials, insecticides, fungicides, herbicides, disinfectants, defoliants, and plant growth regulators. The majority of the references are from the recent publications, within the last 5 years, where the RDI in this area is more intense, however, the pieces of literature available before this period will be also included if they are of some help, so that the content of the paper reflects a general overview of the use of layered hydroxides and layered double hydroxides for pesticide nanodelivery systems.

## 2. Commercially Available Nanoproducts for Agriculture Applications

Various applications of nanotechnology for the agriculture sector have been researched and developed either for their direct or indirect uses, to improve stress tolerance, prolong the shelf life of crop products, etc. These include agro-nanochemicals for fertilizer, soil enhancer, plant protection and management, precision farming, sensors and detection, pesticide remediation, animal production, postharvest management, etc. Among the most active countries in promoting agro-nanotechnology products are India, Germany, UK, USA, Vietnam, Taiwan, Brazil, China, Malaysia and the Netherlands [11].

As a result, more than 230 nanoproducts of 37 types for various agriculture applications were introduced into the global market from 75 companies of 26 countries for animal husbandry, fertilizers, plant breeding, soil improvement and plant protection. The latter includes algaecides, herbicides, biocides, disinfecting agents, fumigants, etc. Among the notable manufacturers are Neufarm GmbH, Plant Vitality Ltd., Kanak Biotech, FRAmelco, Aqua-Yield Hub, Organic Fertilizing, Reed Mariculture Inc, Prodotti Arca S.r.l, Kimitec Group, Richfield Fertilisers Pvt, Blue Planet Environmental, Danaflex Nano, Bioteksa, AgriLife, NanoL and Baltic, Vive Crop Protection, DVS BioLife Ltd., Samarita, and Litho Plant [11]. Examples of nanofertilizer manufacturers are given elsewhere [8]. A list of commercially available nanoproducts and their economic viability can be found elsewhere [12].

## 3. Pesticides: Definition, Classifications and Demand

### 3.1. Definition

Based on the United States Environmental Protection Agency (EPA), a pesticide is defined as any substance or a mixture of substances intended for (i) preventing, destroying, repelling, or mitigating any pest; (ii) plant regulation, defoliant, or desiccant; and (iii) use as a nitrogen stabilizer. Pesticides are usually composed of an active ingredient (AI) to prevent, destroy, repel, or mitigate a pest, or is a plant regulator, defoliant, desiccant, or nitrogen stabilizer. Together with the AI are inert ingredients (II), which are important for product performance and usability.

Many databases have described the basic and other information about AI for pesticides, such as IUPAC, EU, etc. These can be found on their homepages. In addition, a review of the ecotoxicological and regulatory aspects of environmental sustainability of nanopesticides can be found elsewhere [13,14].

### 3.2. Classifications

Pesticides can be grouped under chemical and nonchemical or biopesticides. The former can be organic or inorganic and are classed as Ia, Ib, II, III and U according to their toxicity, indicating extremely hazardous, highly hazardous, moderately hazardous, slightly hazardous and unlikely to present an acute hazard, respectively. This is based on a comprehensive classification of pesticides that can be found on the WHO homepage [15].

The IUPAC pesticides properties database (PPDB) is another comprehensive pesticide database on the chemical identity, physicochemical, human health and ecotoxicological data can be found at the Agriculture & Environment Research Unit (AERU) at the University of Hertfordshire, for a variety of end users to support risk assessments and risk management [16]. Similar to that, the European Union (EU) also has a comprehensive pesticides database which can be accessed [17]. Based on the National Pesticides Information center (NPIC) pesticides are classified according to their target use as given in Table 1 [18].

### 3.3. Demand

Agrochemicals are important for the agricultural sector, involving multi-billion-dollar businesses of various multinational companies in developed and developing countries. This is due to the rapid growth in demand, triggered by various factors especially the growing food demand due to rapid growth of the human population, less efficiency of the input–output balance of the food products, climate factors, etc.

The global demand for agrochemicals was estimated at a value of USD 208.6 billion in 2020, and this value is projected to reach USD 246.1 billion by 2025, at a CAGR of 3.4% during the forecast period. The fungicides group contributes about 9% with a value of USD 18.7 billion and is projected to reach a value of USD 24.5 billion by 2025, growing at a CAGR of 4.6% during the forecast period [1]. The agrochemicals in this category include pesticides (herbicides, insecticides, fungicides), fertilizers (nitrogenous, phosphatic and potassic) and crop application (Cereals & Grains, Oilseeds, Fruits & Vegetables). Herbicide-based agrochemicals contribute the highest demand in the agrochemical sectors due to their convenient use in economical crops such as sugarcane, rice, soybean, oil palm, rubber, cotton, etc.

Bayer and BASF (Germany), Yara International (Norway), Compass Minerals (USA), Syngenta (Switzerland) and Adama Ltd. (Israel) are some of the global players in the agrochemical industry [1].

## 4. Nanomaterials: Definition and General Properties for Nanotechnology Applications

As mentioned earlier, many types of nanopesticides can be generated from various types of nanomaterials; 0D, 1D, 2D and 3D. These including chitosan nanoparticles, various types of carbon nanostructures such as graphene and its derivatives, carbon nanotubes, carbon nanodots, etc. In addition, micelles, chitin, clays, clay derivatives and synthetic clays, activated carbon, zeolites, silica, etc., have also been used for the formation of host–guest nanopesticides.

### 4.1. Definition

Based on ISO/TS 80004, nanomaterials are defined as “the materials with any external dimension in the nanoscale or having an internal structure or surface in the nanoscale”, with nanoscale defined as the “length range approximately from 1 nm to 100 nm”. This includes a discreet piece of material (nano-objects) and materials with internal or surface structure on the nanoscale (nanostructured) [19].

Based on the dimension, nanomaterials can be categorized into 0D, 1D, 2D and 3D. Fullerenes, carbon nanodots, quantum dots, are some examples of 0D nanomaterials. Carbon nanotubes (CNT), nanofibers, etc., are 1D, and clays, synthetic clays, layered hydroxides and layered double hydroxides, layered triple hydroxides, graphene and its derivatives are some examples of nanomaterials of the 2D family. Activated carbons, zeolites and metal-organic frameworks (MOF) are some examples of the 3D family.

### 4.2. General Properties of Nanomaterials

Nanomaterials have superior quality compared to their bulk counterparts’ higher strength and higher surface area to volume ratio, chemical reactivity and conductivity, etc. Products based on nanotechnology are produced due to one or more of these superior properties, and lately are visible in many sectors including agriculture, etc., as discussed earlier. This is due to the belief and proof that these types of products could improve quality of life, either directly or indirectly.

For engineered nanomaterials, their physicochemical properties are usually attached to the general superior properties together with the intended specific properties. For example, due to the high specific surface area, more guests can be loaded onto the surface, and due to the intercalation and deintercalation properties of the guests, ion exchange can take place, resulting in slow- or controlled-release properties of the guests of the host–guest nanomaterials. This will be discussed later.

### 4.3. Toxicity of Nanomaterials

Human beings, animals, plants, and other living organisms, etc., and the environment, are subjected to various nanomaterial exposures from different kinds of their origins; engineered, natural and incidental, from time to time. With the advancement of nanotechnology, especially with new types of engineered nanomaterials being intensely synthesized, there is sometimes a threat to life. Therefore, nanotoxicology; the study of the toxicity of nanomaterials to life and the environment, is equally important, in tandem with the advancement of nanotechnology [20,21].

Previous works have shown that nanofungicides based on Zn/Al-LDH were found to be more potent and safer for seeds and seedlings, compared with their counterparts. In addition, the presence of cations such as zinc from the host improves the growth of the seedling [9]. Similarly, the cytotoxicity, genotoxicity and cell death assays of chitosan-based fungicide nanoparticles did not reveal any cytotoxicity or genotoxicity potential [22]. Although many other previous works have also indicated that encapsulation of pesticides generally reduces their toxicity, this cannot be easily generalized to other nanopesticides. Therefore, research, development and innovation (RDI) in nanotoxicology is as important as its nanotechnology counterparts [23,24,25].

### 4.4. Nanomaterials for Agricultural Practices

Nanomaterial is one of the bases for various nanotechnology applications due to their superior physicochemical properties as mentioned earlier. Together with nanoscience, their physicochemical properties can be engineered to tailor their requirements for various technological applications in various fields which demanded the multidisciplinary kind of RDI.

As previously mentioned, one of the most important nanotechnology applications is in the agricultural sector, including pesticides for plant protection. Due to very diverse nanomaterials that can be used for the generation of new nanopesticides, here we focus our review only on the layered hydroxides; layered hydroxide salts and layered double hydroxides. This is due to their special reversible intercalation and deintercalation process, their 2D inorganic layered structure enables them to host various active ingredients, the pesticide active agents. By the virtue of the ion exchange properties of the host–guest complex, these properties have been exploited for the controlled-release formulation of pesticides. This will be discussed later.

## 5. Layered Hydroxides

Layered compounds are composed of two-dimensional units connected to each other by weak forces. Layered hydroxide salts (LHS) with a general formula of M^2+^(OH)_2−x_(A^m−^)_x/m_·nH_2_O) and layered double hydroxides (LDH) with the general formula of M^2+1−x^M^3+x^(OH)_2_(A^m−^)_x/m_·nH_2_O) are layered compounds, where the 2D inorganic metal hydroxides sheets are stacked together by weak forces.

**Figure 1:**
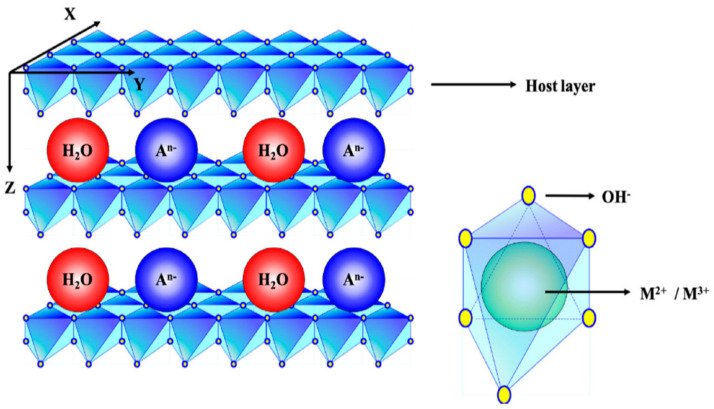
The structure of LDH is composed of a 2D, inorganic, brucite-like structure. Layered hydroxide has a similar structure, but only composed of M^2+^ instead of M^2+^/M^3+^ in the LDH. The counter anion, A^n-^ together with water molecules are present in the inorganic interlayer [26].

Layered hydroxides and layered double hydroxides are examples of 2D layered materials in this family. The former has the brucite-like structure, where the inorganic layer is composed of only one cation (M^2+^) such as Mg or Zn, etc., while for the latter, the inorganic layer is composed of at least two cations; M^2+^ and M^3+^, such as Mg with Al, Zn with Al, etc. (Figure 1). Due to its positively charged inorganic layers, counter anions are intercalated in between or interlayer of the 2D layered structure to keep the material neutrally charged. This anion can be exchanged with various anions for various applications, especially for agrochemicals such as pesticides, fungicides, etc.

Layered hydroxides (LHs) and layered double hydroxides (LDHs) are widely used for the formation of a new generation of agrochemicals and other uses related to agriculture, based on nanotechnology platforms. As shown in Figure 1, these two families of LHs and LDHs are used to synthesize smart fertilizers, nanosensors, precision farming, pesticide remediation, nanopesticides and other uses, either directly or indirectly.

The 2D, inorganic, layered structure is composed of single or double metal ions. The thickness of this inorganic layer is well established to be 4.8 A. The basal spacing of the LDH with nitrate, sulfate, carbonate, chlorine and iodine is around 7–9 A, depending on the counter anion occupied in the interlamellar of the LDH [27]. Upon intercalation of the guest anions such as pesticides, drugs, etc., this basal spacing will be expanded, depending on the size and spatial orientation of the anions in the interlamellar of the LDH [28].

Various active ingredients, anions, organic and inorganic species can be intercalated in the LDH interlamellar as the guests using the supramolecular chemistry approach. As a result, various applications can thus be generated. The nanostructured LDH has been used as the host of the AI, and the resulting complex inherited superior properties of the host; controlled release, being more potent, having higher bioavailability, etc. [9].

### 5.1. Application of Layered Hydroxides

**Figure 2:**
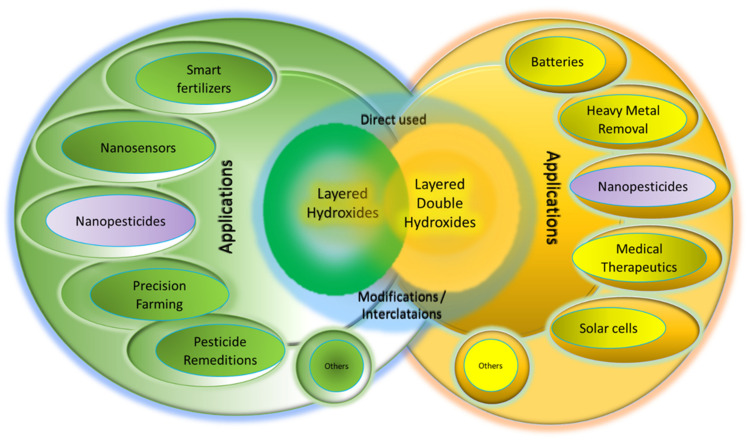
Various technological applications of LHs and LDHs either as they are or after modification by intercalation of AIs using the host–guest supramolecular chemistry approach.

LHs and LDHs are composed of positively charged inorganic layers (Figure 1), and the counter anion can be replaced by various AIs for various purposes as shown in Figure 2. These include nanopesticides for plant protection [29], drug delivery [30] and gene delivery [31], sunscreen protection [32], catalysis [33] and environmental remediation, [34], supercapacitors [35], cosmetics [36], etc. If drugs or therapeutic agents are intercalated into the interlayers, various nanodrugs [37], or nanotherapeutic delivery systems can be generated which are useful for anticancer purposes [38], anti-TB purposes [39], etc. By virtue of the ion-exchange properties or dissolution of the inorganic brucite-like layers (or both), the intercalated AIs can be released from their hosts—LHs or LDHs—resulting in the controlled-release properties and will be discussed in Section 5.2.

As mentioned earlier, LHs and LDHs are composed of inorganic, brucite-like structures which, upon calcination at a certain temperature, result in the collapse of the 2D layered structure and subsequently generate various single or mixed oxides, respectively. This results in a uniform distribution of oxide which is useful for catalyst [33] purposes. Due to their high ion-exchange capacity, LDHs can be used for the remediation of heavy-metal-contaminated or pesticide-contaminated water [34]. Other applications, including smart fertilizers, nanosensors, etc., are shown in Figure 2.

### 5.2. Application of Layered Hydroxides in Agriculture

**Figure 3:**
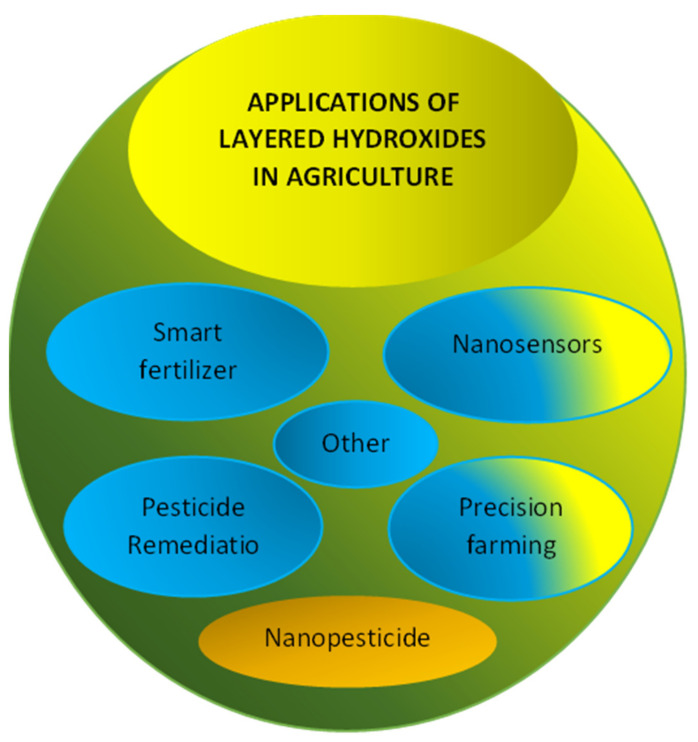
Various applications of layered hydroxides in agriculture.

Various applications of layered hydroxides in agriculture are summarized in Figure 3; smart fertilizers, nanosensors, pesticides remediations, precision farming, nanopesticides, etc. LHs and LDHs can be used in various agriculture sectors either as their original synthetic compounds or their derivatives; after modification, intercalation, etc., [5,6,8,12,40].

One of the important applications of LHs and LDHs is the formation of a new generation of pesticides. Various types of pesticides; herbicides, insecticides, fungicides, etc., as the AIs can be intercalated into the LHs and LDHs hosts. The number of combinations of host–guest complexes that can be formed via supramolecular chemistry is huge, thus opening up unlimited opportunities to produce and explore this area of research, development and innovation.

### 5.3. Supramolecular Host–Guest Chemistry

Supramolecular chemistry is the chemistry of molecular assemblies of intermolecular noncovalent bonds that result from the association of two or more chemical entities. The binding of the chemical entities can be from various weak bonds such as hydrogen bonds, Van der Waals, hydrophobic, etc., or even fairly strong bonds such as metal-ligand coordination or electrostatic bonds [41,42].

Host–guest chemistry is the key enabler in constructing various nanomaterials for various applications from agriculture to medicines and from physics to engineering. Currently, supramolecular chemistry is truly interdisciplinary, especially in the nanotechnology era, where research in this area requires various expertise in the areas of biology, biochemistry, chemistry, material science, physics and engineering, etc. Various types of host–guest nanomaterials can be generated using supramolecular chemistry. For example, complex biological structures can be produced by molecular self-assembly, where a spontaneous association of stable molecules via noncovalent bonds results in a well-defined aggregate [43]. 

One of the important factors for the formation of host–guest supramolecular structures is the interaction between the host and the guest via various processes such as attachment, entrapment, encapsulation, intercalation, adsorption, conjugation, or their combinations. This is very much determined by the type of physicochemical properties of the host and the guest and the bond that can be formed between them.

The types of bonds that will be formed between the host and the guest can be from weak bonds such as Van der Waals, hydrogen bonds, hydrophobic forces, etc., or even strong bonds such as metal-ligand coordination or electrostatic bonds.

**Figure 4:**
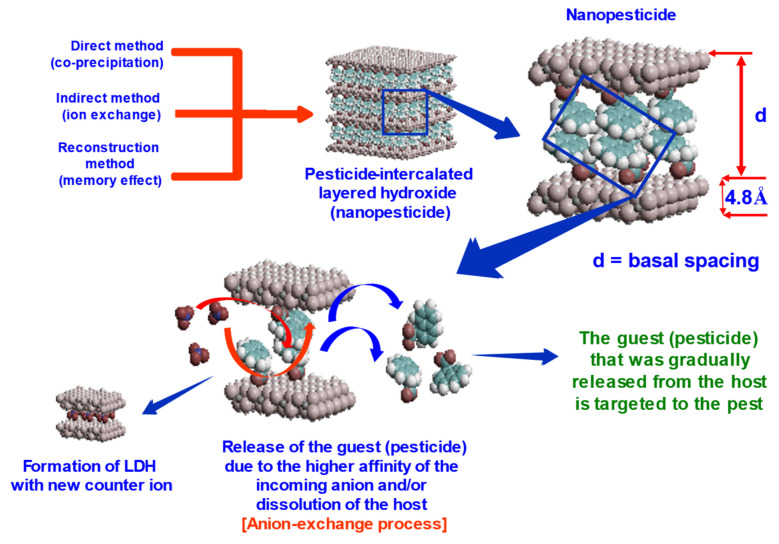
Layered double hydroxide inorganic 2D structure, the formation of the nanopesticides by adopting host–guest supramolecular chemistry, and the release of the guest from the host via the ion exchange process, together with the dissolution of the host and the possible formation of the “new” LDH with the new incoming anions that are available in the surrounding environment.

Nanopesticides can be synthesized using host–guest supramolecular chemistry using LHs or LDHs 2D inorganic layered structure as the host and the AI pesticide as the guest. The formation of the resulting nanopesticide is called a “complex”, which refers to the nanopesticides that were formed. The term “intercalation” is usually referred to the encapsulation of the AI into the 2D inorganic layered structure (Figure 4). This can be confirmed by various analytical techniques, and some of the important ones are given in Table 2. 

Figure 4 shows a schematic diagram of how layered double hydroxide can be used for the formation of the nanopesticides by adopting host–guest supramolecular chemistry using different approaches; direct co-precipitation, indirect ion exchange and reconstruction, or memory effect methods. Following the formation of the nanopesticides, the release of the guest (the pesticide or the AI) from the host is accomplished either via the ion exchange process, the dissolution of the host or via both. The AI that was released is targeted to the pest. Depending on the surrounding environment, the formation of the “new” LDH with the new incoming anions that are available in the surrounding environment is possible. This can be verified by various analytical techniques, especially the XRD.

### 5.4. Method of Synthesis

There are three main methods that can be adopted to synthesize nanopesticides using host–guest supramolecular chemistry, namely direct, indirect and reconstruction or “memory effect”. The direct method involves the co-precipitation of the anions and cations in a controlled pH environment. In the indirect method, the counter anion that already exists in the interlayers of the LDH is exchanged with the AI as the guest from the solution. This can be achieved by placing the preprepared LDH into a solution containing the anion of the pesticide AI so that the anion exchange process will take place, resulting in the intercalation of the new guest, which is the AI for the formation of the host–guest nanomaterial [44,45,46,47,48].

The host–guest supramolecular complex can be also synthesized using the regeneration or the “memory effect”. For example, LDH was prepared first and then calcined at a certain temperature to produce a mixed oxides phase. Rehydration by contacting the calcined LDH with an aqueous solution containing anions to be intercalated results in the intercalation of the anion into the LDH lamellar, and this process is called “memory effect”. Other methods such as sol–gel, solid hammer mill, micelles-assisted, etc., can be also adopted and the details of these processes can be found elsewhere [49]. 

### 5.5. Physicochemical Characterizations

In order to confirm whether the host–guest supramolecular complex was formed after the synthesis of the nanopesticides, various physicochemical analyses have to be performed and are only discussed briefly here. Table 2 summarizes the techniques used and the information that can be gathered from them.

The XRD is an excellent technique to confirm the intercalation episode of the guest (pesticide active agent) into the inorganic host (LHs or LDHs). Of course, other supporting techniques are also important to support it. For example, work on the synthesis of nanofungicide using hexaconazole as the AI into the Zn/Al-LDH interlamellar, resulted in the expansion of the basal spacing from 8.72 to 28.87 Å, due to the size and spatial orientation of the guest, with hexaconazole compared to nitrate species in the LDH. In addition, four harmonic reflections can be also observed. A slow scan revealed that eight harmonics reflections can be resolved, resulting in a better basal spacing of 29.42 Å, an average from the eight harmonics [9]. 

**Figure 5:**
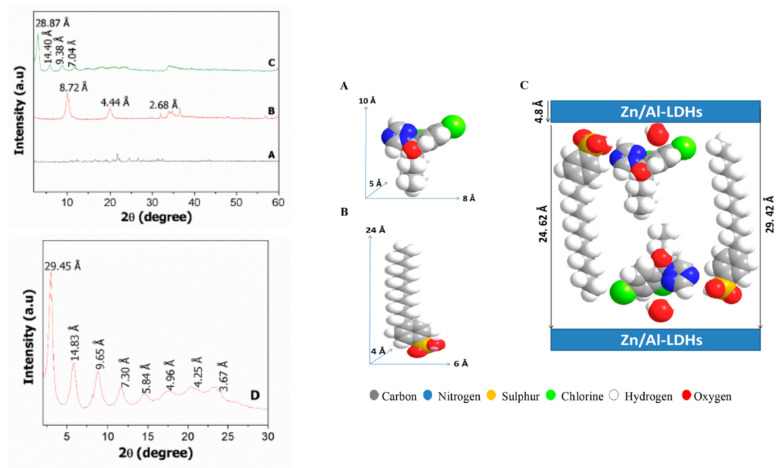
XRD patterns of free hexaconazole (A), Zn/Al-LDH (B), and hexaconazole-intercalated Zn/Al-LDH (the nanofungicide) (C), and the slow scan with a dwell time of 0.5°/min (D) showing details of the eight harmonics, and from them the average value of the interlamellar (nxd) was calculated to be 29.42 Å (left figure) and the 3D structure of hexaconazole (**A**), sodium dodecylbenzenesulfonate (**B**) and plausible arrangement of hexaconazole and sodium dodecylbenzenesulfonate in the intergalleries of HZALDH nanocomposite (**C**) (right figure) [9].

Using the software, the three-dimensional structure of the guest molecule, hexaconazole, can be obtained. It is known that the Zn/Al-LDH brucite thickness is 4.8 Å, therefore the space available for the spatial orientation of the organic moiety, hexaconazole, is 24.62 Å. Therefore, the plausible spatial orientation, taking into account the co-presence of sodium dodecylbenzene sulfonate, is as given in Figure 5 [9].

### 5.6. Controlled-Release Properties

One of the most important properties of the host–guest supramolecule based on the 2D inorganic layered structure is its ability to release the guest gradually if there exists an external force in the surrounding environment. For example, in the ion exchange process, due to the higher affinity of the incoming anion, the AI that was intercalated will be replaced by it, resulting in the AI being released as the outgoing anion (Figure 4).

For the in vitro study, the nanopesticide is exposed to a buffer solution of certain pH and the amount of the guest released is measured at a preset time, using analytical techniques such as high-performance liquid chromatography (HPLC) or UV–visible spectroscopy. A plot of cumulative release against time can be obtained. The linear fit of the amount of the released guest is then fitted to mathematical models, where the R^2^ value together with the plot will help in the selection of the best mathematical model that fits the kinetic release of the guest, the AI.

### 5.7. Kinetic Release of the Guest from the Nanopesticides

Pesticide release is a process where AI leaves the host and eventually becoming available for the biological action. This study involves AI release rate, dissolution, diffusion, erosion and the study of the factors that affect the release rate. In vitro releases of AI into a solution under a certain condition are usually used to study the release of the AI from the synthesized nanopesticides [50].

There are many mathematical models to be used that fit the release of the AI from the host into the solution. Buffer solutions of certain pH are usually used to mimic the physiological environment of the target usage. Among the models used are Fick’s First law, Fick’s second law, zeroth-order, first-order, second-order, Hixson–Crowell, Korsmeyer–Peppas, Wiebull, Higuchi, the Baker–Lonsdale model, the Hopfenberg model, etc. [51,52,53,54,55,56,57,58]. This is summarized in Table 3.

By looking at the mathematical models, from the best-fitted model of the release of the AI from the host we can deduce the mechanism of the release. For example, if the release of the AI is governed by the Hixson–Crowell Equation, then the mechanism for drug release is by dissolution, involving changes in surface area and diameter of the nanoparticles, and so the release mechanism is not by diffusion. The details of these mathematical models can be found elsewhere [51,52,53,54,59,60].

## 6. Nanopesticides Synthesized Using 2D Inorganic Layered Hydroxides Hosts

For the 2D inorganic layered hosts, most of the RDIs are focusing on the layered hydroxides. Therefore, our review is based only on three types of hosts, namely zinc-layered hydroxide, magnesium/aluminum-layered double hydroxide and zinc/aluminum-layered double hydroxide, together with other LDHs.

### 6.1. Zinc Layered Hydroxide

LH has a 2D inorganic layered structure, which is composed of a single metal hydroxide. Zn-LH is the most common one used to host various AI guests for the synthesis of nanopesticides that can be found in the literature, as shown in Table 4. For example, isoprocarb and carbamate were separately intercalated into the Zn-LH for the formation of their nanoinsecticides, resulting in better thermal stability and environmental friendliness of the AI compared with their counterparts [61,62].

Other pesticides that were intercalated into Zn-LH are 3-(4-methoxyphenyl) propionic [63], imidacloprid [64], 4-(2,4-dichlorophenoxy) butyrate and 2-(3-chlorophenoxy) propionate [65] and 2-methyl-4- chlorophenoxyacetic [66]. They were synthesized due to their superior qualities compared with their counterparts.

A pesticide, 3-glycidyloxypropyl trimethoxysilane was intercalated into the Zn-LH host for the formation of its nanopesticides. The release of the AI was found to be governed by the pseudo-second-order and parabolic-diffusion models [67]. Sodium dodecyl sulfate [68] and chlorpyrifos [69] also were intercalated separately into the LH host either directly (for the former) or indirectly using a surfactant such as dodecylbenzenesulfonate in the latter. Both of them were claimed to be more superior compared to their counterparts.

As shown in the table, advantages such as being environmentally friendly, improved thermal stability and controlled-release properties are the superior qualities obtained when the host–guest complex nanopesticides were formed from their AI counterparts. This is why these type of nanopesticides are worth exploring as they provide many benefits.

### 6.2. Zinc/Aluminum-Layered Double Hydroxide

Zn/Al-LDH is subjected to more intense research compared to other LDHs, regarding its use as the host of various AI pesticides for various nanopesticide applications. This is because it is relatively simple and cheap to prepare, and it is easier to form the 2D, well-ordered, inorganic, layered structure with good crystallinity.

Table 5 shows the nanopesticides prepared using host–guest supramolecular chemistry based on Zn/Al-LDH. AIs such as mandelic acid were intercalated into Zn/Al-LDH to be used as slow-release preservatives and for crop protection [70]. Herbicides or defoliants; 2,4-dichlorophenoxy and 4-chlorophenoxy acetate were simultaneously intercalated into the Zn/Al-LDH and were found to be promising candidates which can simultaneously control the release of the two AIs at differently controlled rates [71]. Similarly, 2,4-dichlorophenoxyacetic acids in the presence of glyphosate showed better herbicide properties [72].

Quinclorac-intercalated LDH is more stable than its free AI form, and can be used as a nanopesticide for rice crop protection [64,74]. Carbonate was used as the guest, and the nanopesticide formed was found to be a better alternative for an antimicrobial agent than its counterpart [73].

The fungicides hexaconazole and dazoment were intercalated separately into the Zn/Al-LDH, and the resulting nanofungicides were found to be more potent than their AIs in treating basal stem rot disease of oil palm trees, one of the important economical crops of palm oil producers such as Malaysia, Indonesia, etc. In addition, the presence of Zn from the inorganic layer helps the early stage growth of the young oil palm [75]. Their superior quality is believed to be due to the controlled-release property, resulting in better bioavailability and less toxicity, due to the deintercalation of the AIs into the LDH interlayers by the ion-exchange process.

Zn/Al-LDH-nalidixic acid nanopesticide is used to control pest infestation and plant diseases [76], increasing the effectiveness of pest control and minimizing possible soil and water contamination [77]. This organic biocide was found to be very effective, which can eliminate almost completely bacteria within a short time. Similar properties were also observed for pipemidic acid-intercalated Zn/Al-LDH [78]. Furthermore, 4-(2,4-Dichlorophenoxy) butyrate nanohybrid [90] and 4-chlorophenoxyacetic acid [91] were also used as the guests for the synthesize of nanopesticides. In the former, the organic moiety can be more easily released and the release property of the guest can be tuned up. In the latter, it provides an alternative for low-cost and green herbicides.

Defoliants are chemicals that are adsorbed by broad-leafed plants and kill them due to excessive hormonal growth. Organic active agents such as 2,4-dichlorophenoxyacetic acid and 2,4,5-trichlorophenoxyacetic acid, tribufos, dimethipin, and thidiazuron are examples of chemicals that are used as defoliants. These compounds are used to selectively kill weeds and unwanted plants. They are also helpful for effective harvesting of certain crops, such as cotton, etc. Research on nanodefoliants with controlled-release properties was achieved by the intercalation of the herbicides 2-(2,4-dichlorophenoxy) acetic acid, 2-methyl-4-chlorophenoxyacetic acid (MCPA) and 3,5-dibromo-4-hydroxybenzonitrile (bromoxynil) into the Zn/Al-LDH nanosheets by a modified co-precipitation method. The nanodefoliants formed were found to reduce the volatilization by 3-fold and retard leaching through the soil, simultaneously [79].

Avermectin is used in agriculture and horticulture to control insect pests on many crops and ornamentals. Its intercalated product using the Zn/Al-LDH as the host shows better water-dispersible controlled-release formulation [80]. Potent antimicrobial activity against *Lactobacillus* in a short period, better production and better stability were obtained using Bacteriocin avicin [81]. Benzoate and its derivatives were also subjected to an intercalation process, where the AI was released very slowly, resulting in the rate of killing being depending on the derivatives used [82].

Carboxymethyl cellulose-intercalated Zn/Al-LDH nanopesticide offers a better agro-nanochemical to reduce the excessive usage of herbicide in paddy cultivation [83], while its chitosan counterpart could enhance the antimicrobial effect compared to its AI [84]. Similar work on chlorpyrifos/cyclodextrin indicated the slow-release property, which is due to the different arrangements of cyclodextrin in the resulting synthesized material. In addition, the release rate of the AI from nanohybrids was pH-dependent [85].

Cinnamate anion-intercalated Zn/Al-LDH gives green protective coatings for crops’ protection [86,87,92]. Intercalated ciprofloxacin, sulfanilamide, and oxazolidinone, and the nanopesticide products were found to improve the antimicrobial properties of the AIs.

The intercalation of β-cypermethrin in sulfonated hydroxyethyl-β-cyclodextrin and sulfobutyl ether β-cyclodextrin resulted in nanopesticides, in which the release rate of the AIs can be tuned using the pH [88]. The work on the intercalation of a natural product such as Eucalyptus oil is very promising, where the amount of the effective AI can be reduced to very low concentrations, able to kill *Staphylococcus* sp. and inhibit *Pseudomonas aeruginosa* growth [89]. Last but not least, pectin/cinnamate acid-intercalated Zn/Al-LDH resulted in dual applications; cosmetics and crop protections [86].

As shown in the above table, the advantages of the superior qualities when the host, Zn/Al-LDH, was used to guest the AIs for the formation of the nanopesticides are similar to the zinc layered hydroxide (ZLH) host, but the latter is much easier and cheaper to prepare because only one metal cation, Zn^2+^ is used for the formation of the inorganic, 2D layered structure. In addition, based on the previous studies, the presence of the Zn^2+^ could help the growth of the seedling [9,75]. Therefore, the Zn/Al-LDH not only acts as the host but also serves to supply micronutrients to the crops.

### 6.3. Magnesium/Aluminum-Layered Double Hydroxide

Magnesium/aluminum-layered double hydroxide (Mg/Al-LDH) is in the family of LDHs where the inorganic 2D layered structure is composed of Mg^2+^ and Al^3+^ cations and this inorganic sheet is positively charged. The counter anions are inserted into the intergallery of the Mg/Al 2D inorganic structure to balance the charge so that the resulting materials are neutrally charged.

Many AIs can be used as the counter anions as shown in Table 6. The choice is very much dependent on the final target use and the different desired advantages of the properties of the nanomaterial, LDH, such as controlled properties, higher thermal stability, etc. Herbicides such as (4-chloro-2-methylphenoxy) acetic acid [93], 2,4-dichlorophenoxyacetic acid [94], 2-methyl-4-chlorophenoxyacetic acid [95] and atrazine [96] were used for the intercalation process so that the resulting nanoherbicides formed have slow-release properties, which then reduce the environmental hazards. Other special properties of these nanoherbicides are given in Table 6.

Ciprofloxacin is fluoroquinolone antibiotic, broad-spectrum antimicrobial and is an important class of drugs for both human and animal health [103]. Its nanopesticide complex with Mg/Al-LDH gave higher thermal stability and other CRF properties, resulting in its superior quality compared with its counterpart [97].

Cinnamic acid is a plant growth regulator (PGR) in the cis form [104] but it is an anti-auxin in its trans form [98,105]. Both of them were separately intercalated into the Mg/Al-LDH and the resulting nanopesticide can be used as a green pesticide based on its required effect.

Hexaconazole and triadimenol are fungicides used to control various fungal diseases. The former can be used to treat basal stem rot disease in oil palm trees [9] and the latter is used as a fungicide for pineapple pine seedlings, Christmas trees, residential (sod farm) and commercial turf, ornamentals, and landscapes. It is also used as a seed treatment on barley, corn, cotton, oats, rye, sorghum, and wheat [106]. These two AIs were found to form their Mg/Al-LDH complexes where the percentage loading of hexaconazole is higher than triadimenol [99].

Imazamox is an herbicide to prevent plant regrowth and emergent vegetation. Unfortunately, the repeated use of this herbicide with the same mode of action can lead to herbicide-resistant plants [107]. It is generally safe for nontarget species, including humans, but the possibility of toxicity through interfering with other biochemical pathways cannot be ruled out. Intercalation of imazamox will decrease water-pollution risk and maintain efficacy with the advantages of soil compatibility [100].

As shown in Table 4, other AIs useful for plant protection such as camphorsulfonic acid [97], long-chain (C18) unsaturated fatty acid anions; elaidate, oleate, and linoleate [101], sulfamerazine and salicylaldehyde [102] were also subjected to the intercalation into the Mg/Al-LDH nanosheets for the formation of their respective complexes for the obvious reason; the superior physicochemical properties of the host–guest nanomaterials, especially the CRF properties.

Similar to ZLH and Zn/Al-LDH as the hosts, the Mg/Al-LDH host could provide micronutrients, magnesium to promote crop growth, and at the same time acted as the nanopesticide for plant protection, in addition to its superior qualities. These bifunctional properties are important for high throughput agricultural practices.

### 6.4. Other Layered Double Hydroxides

Apart from Zn/Al-LDH and Mg/Al-LDH, other layered double hydroxides were also used as the hosts for different AIs such as Co/Cr-, Mg/Fe-, Mg/Al/Fe-, Zn/Cr- and Zn/Cu-LDH (Table 7). For example, haloxyfop and hexaflumuron were intercalated into the Co/Cr-LDH for the formation of nanoinsecticides [108]. A herbicide, 4-chloro-2-methylphenoxy) acetic acid, was intercalated into Mg/Fe- and Mg/Al/Fe-LDH for the slow release of the herbicide to reduce the hazardous effects that it can pose to the environment [93].

Zn/Cu-LDH also was used to host the biocides zinc pyrithione and copper pyrithione for the formation of nanopesticides with lower toxicity compared to their free forms. The nanopesticides were found to be a good candidate for antifouling applications [109]. The same host was also used for the intercalation of hydroxycinnamate where the orthoisomer showed a more sustained release compared to the other isomers [110].

According to Ragavan et al., 2006 [111] 4-chlorophenoxyacetate, 2,4-dichlorophenoxyacetic and 2,4,5-trichlorophenoxyacetate were intercalated into (LiAl_2_(OH)_6_) Cl·*x*H_2_O LDH ((Li–Al–Cl) LDH) by the ion-exchange method. The intercalation and deintercalation of the AIs were found to be dependant on the type of chlorophenoxyacetic acid used.

Algaecides are active agents that kill or prevent the growth of algae. So far, not many works on nanoalgaecides can be found in the open literature. It was indicated that Zn/Fe-LDH can be used as a photocatalyst under visible light for *Microcystis aeruginosa* inhibition, without the requirement of any active agent [40].

The use of various M^2+^ and M^3+^ for the formation of the inorganic, 2D, layered structure of the LDHs to host the AIs is beneficial because it promotes crop growth, where the M^2+^ and M^3+^ could provide micronutrients after the release of the AIs from the LDH interlayer. Again, the resulting nanopesticides are expected to have bifunctional properties which are important for high yield in modern agriculture.

## 7. Practical Applications, Future Research Perspectives, Challenges and Limitation

Based on the works of literature, it is clear that nanopesticides generally offer superior qualities, which are more potent, more economical, and user and environmentally friendly compared with their conventional counterparts, and therefore have promising practical applications. This is due to the fact that the same agricultural practice can be adopted, but we simply switch the conventional pesticides to nanopesticides. In addition, the synthesis of nanopesticides using the host–guest supramolecular chemistry approach is relatively simple and cost-effective, where the same commercially available AIs can be used as the guests. The cost for transforming conventional pesticides to their nanopesticides counterparts is outweighed by their benefits, and put together with their superior qualities to the human life and the environment, surely nanopesticides have promising practical applications in the agricultural sector.

The current RDIs are focusing more on single guest AI and only a few works have attempted on the simultaneous intercalation of two AIs for bifunctional nanopesticides. Multifunctional nanopesticides are the way forward, in parallel with the demand of the current high-throughput agricultural practices. In addition, multimodal agro-nanochemicals where macro- and micro-nutrients can be combined with plant protection agents are also important to achieve holistic, efficient crop management. Although findings within the literature for a product at the laboratory scale seem promising, the commercialization usually has to follow several steps, upscaling, field studies, etc. Upscaling of the production of the nanopesticides is another challenge in terms of reproducibility, quality assurance and cost effectiveness. This is another stage of RDI of new products in which financial support is crucial.

Many of the AIs of pesticides are classified under the regulations for use in their agricultural practices and the regulations are usually different for different countries. Again, RDI is important from seed technology to the nursery, and from field studies to postharvest. For the field studies, residual and efficacy analyses are important, where together with nanotoxicology data they are important to obtain the license of commercialization in terms of production, storage, selling and use for the agriculture sector. For commercialization, intellectual property is important to protect the new product from being copied by other parties. Due to related costs of legal fees, etc., which are quite expansive, sometimes this becomes another limitation against the new nanopesticides becoming available at the commercial scale.

Another challenge of a new product is the general acceptance of the public or the planters to switch from their existing familiar product to a new one. Establishing the product in the market via advertisement, etc., is another challenge that is not part of the scientific work but has to be done.

## 8. Way Forward

In this review, we have discussed the current scenario of the research, development and innovation (RDI) for the up conversion of various currently available active ingredient of pesticides for plant protection through nanotechnology platforms using various 2D, inorganic, nanolayered materials; layered hydroxides and layered double hydroxides. The findings from the literature agreed that the resulting nanopesticides have superior qualities compared to their counterparts, and therefore the efforts towards the generation of new agro-nanochemicals which are superior to their conventional counterparts are worth the effort.

Although the RDIs in these areas were quite extensive, not all the toxicology issues, the toxicology benchmark or standard technical characterization in determining the toxicity of the nanomaterials used for host–guest supramolecular chemistry were cared for. Therefore, the nanotoxicology of the nanopesticides is the next direction we shall focus on.

Due to vast types of pesticide active ingredients available in the market that can be used as the guest for the generation of nanopesticides, generalization on their toxicity cannot be easily made. Therefore, it is undeniable that many negative impacts are still underexplored. This includes the toxicity impact of the AIs, as well as the host, and the resulting nanomaterials when they form nanopesticides. Therefore, RDI should be continued and at the same time work on the strategies of integrating the knowledge and sharing them towards comprehensive databases and standards is required, so that these new generations of agrochemicals can benefit not only the agricultural industries but also the user and the environment.

## 9. Conclusions

This manuscript reviews the use of various 2D, brucite-like inorganic layered structures, namely layered hydroxides and layered double hydroxides, to hosts various types of pesticide active ingredients for the formation of various nanopesticides for plant protection. Among them, zinc layered hydroxides and zinc/aluminium-layered double hydroxide were found to be the most popular choices to be used as the host, presumably due to their relative ease to prepare and cheap cost. Other hosts including Mg/Al-, Co/Cr-, Mg/Fe-, Mg/Al/Fe-, Zn/Cr- and Zn/Cu-LDH were also used. The type of AIs intercalated as the guests were found to be wide-ranging, from fungicides to herbicides and from defoliants to plant growth regulators. As the preparation step is fairly straightforward, this is only a step further, where the same AIs of agrochemicals can be used, and therefore the processing cost can be outweighed by the benefits. These structures are more economical, have better efficacy, and are safer to the life, human beings and the environment. This enables us to move on from conventional agrochemicals to a safer, new generation of agrochemicals, “agro-nanochemicals”, produced using nanotechnology platforms. This looks towards the holistic management of pesticides for crop protection using nanotechnology platforms for high-throughput agricultural products.

## Figures and Tables

**Table 1:** Types of pesticides based on the National Pesticides Information Center [18].

Pesticide	Target/Use
Algaecides	Killing and/or slowing the growth of algae.
Antimicrobials	Controlling germs and microbes such as bacteria and viruses, etc.
Biopesticides	Made of living things, come from living things, or are found in nature.
Desiccants	Drying up of living plant tissues.
Defoliants	Causing of plants to drop their leaves.
Disinfectants	Controlling of germs and microbes such as bacteria and viruses
Foggers	Killing of insects that are in the open and touch the pesticides.
Fungicides	Control of fungal problems such as molds, mildew, and rust.
Herbicides	Killing or inhibiting the growth of unwanted plants, aka weeds.
Insecticides	Insect control.
Insect growth regulators	Disrupting the growth and reproduction of insects.
Miticides	Control of mites that feed on plants and animals
Molluscicides	Control of slugs, snails and other mollusks.
Mothballs	Killing of fabric pests by fumigation in sealed containers.
Natural and Biological Pesticides	Control of pests using things found in nature, or manmade versions of things found in nature.
Ovicides	Control of eggs of insects and mites.
Pheromones	Biologically active chemicals which are used to attract insects or disrupt their mating behavior. The ratio of chemicals in the mixture is often species-specific.
Plant Growth Regulators	Altering the growth of plants. For example, they may induce or delay flowering.
Repellents	Repelling unwanted pests, often by taste or smell.
Rodenticides	Killing of rodents such as mice, rats, and gophers.
Synergists	Make certain pesticides more effective, but they are not effective when used alone

**Table 2:** Some important physicochemical characterizations of the nanopesticides synthesized by host–guest supramolecular chemistry.

Techniques	Information on The Physicochemical Properties Obtained
Powder X-Ray Diffraction (PXRD)	Crystallinity, basal spacing, the intercalation of the host into the guest, the plausible spatial orientation of the guest in the interlamellar of the host
UV–Visible Spectroscopy (UV–vis)/High-Performance Liquid Chromatography (HPLC)	Pesticide loading and loading efficiency of the guest, kinetic release of the pesticide from the nanopesticides
Thermal Analysis (TGA/DTG)	Thermal degradation behavior of the nanopesticides
Fourier-Transform Infrared Spectroscopy (FTIR)	Chemical bonds or functional groups formed between the host and the guest
Surface Area Analyzer	Surface area and pore-size distribution of porous nanopesticides
Dynamic Light Scattering (DLS)	Particles’ size and the distribution of the nanopesticides
Field-Emission Scanning Electron Microscope (FESEM)	Surface morphology of the nanopesticides and their distribution
High-Resolution Transmission Electron Microscope (HRTEM)	The internal structure of the nanopesticides and their distribution
Direct Injection Mass Spectroscopy (DIMS)	To confirm the presence of the guest in the interlamellar of the LH or the LDH hosts.
Others	Techniques such as X-Ray Photoelectron Spectroscopy (XPS), Selected Area Electron Diffraction (SAED), Raman Spectroscopy, Atomic Force Microscopy, (AFM), Small-Angle X-Ray and Neutron Scattering Spectroscopy (SAXS/SANS), etc., are also used to support that the guest is intercalated into the host for the formation of host–guest nanomaterial.

**Table 3:** A few mathematical models and equations which are often used for controlled-release and kinetic-release characteristics of the AI from the host into the solution.

No	Mathematical Model	Equations	Denotations	Source(s)
1	Zeroth-order	C = C_0_ − K_0_ t	C = Amount of drug released C_0_ = Initial amount of drug in solution K_0_ = Zeroth-order rate constant t = Time	[55]
2	First-order	dC/dt = −KC	K = First order rate constant	[55]
3	Second-order	1 − (M_t_/M_0_)/t = kt^−1/2^ + b		[58]
4	Hixson–Crowell	C_0_^1/3^ − C_t_^1/3^ = Kt	C_t_ = Amount of drug released in time, t C_0_ = Initial amount of drug in table K = Rate constant	[51,55]
5	Fick’s first law	J = −D_f_ dc/dx	J = Amount of substance passing perpendicularly through a unit of surface area per unit of time D_f_ = Diffusion coefficient dc/dx = Concentration gradient	[56]
6	Fick’s second law	$\frac{\partial \varphi }{\partial t}=D\;\frac{\partial 2\;\varphi }{\partial \;x2}$	*φ* = Concentration in mol/m^3^ *φ* = *φ*(*x*,*t*) is a function that depends on location *x* and time *t* *D* = Diffusion coefficient in m^2^/s	[56]
7	Korsmeyer–Peppas	F = (M_t_/M) = K_m_ t^n^	F = Fraction of drug release time M_t_ = Amount of drug release time M = Total amount of drug dosage K_m_ = Kinetic constant n = Diffusion or release exponent t = Time	[55]
8	Wiebull	$f\left(x,\;\lambda ,\;k\right)\;=\left\{\begin{array}{c} \frac{k}{\lambda }{\left.\left(\;\frac{x}{\lambda }\right.\right)}^{k-1}e^{-\left(\frac{x}{\lambda }\right)k}\;\;\;\;\;x\;\geq \;\;0,\\ \;0\;\;\;\;\;\;\;\;\;\;\;\;\;\;\;\;\;\;\;\;\;\;\;\;\;\;\;\;\;\;\;\;\;\;x<0 \end{array}\right.$	K < 1 = Failure rate decreases over time K = 1 = Failure rate is constant over time k > 1 = Failure rate increases over time	[57]
9	Higuchi	Q = K_H_ t ^1/2^	Q = Cumulative amount of drug released at the time, t K_H_ = Higuchi constant t = Time	[52]
10	Baker–Lonsdale	F_1_ = 3/2(1 − (1 − C_t_/C_∞_)^2/3)^ C_t_/C_∞_ = kt	C_t_ = Drug release amount at time, t C_∞_ = Amount of drug released K = Release constant	[53,55]
11	Hopfenberg	Q_t_/Q_∞_ = 1 – (K_0t_/C_0a0_)	Q_t_ = Amount of drug released in time, t Q_∞_ = Amount of drug dissolved when the dosage form is exhausted C_0_ = Initial concentration of the drug A_0_ = Initial radius sphere for a slab	[54]

**Table 4:** Nanopesticides synthesized using host–guest supramolecular chemistry using zinc-layered hydroxide as the host and various AIs as the guest for various targets and advantages.

AI Guests	Target/Advantages	Source(s)
Isoprocarb	A mesoporous-type material forming an environmentally friendly insecticide	[61]
Carbamate	A significantly improved thermal stability of the anion compared to its pure form.	[62]
3-(4-Methoxyphenyl) Propionic	Can be synthesized using ion exchange and co-precipitation methods for the formation of a new generation of agrochemicals.	[63]
Imidacloprid	Good controlled-release properties, overcoming the excessive usage of insecticide in paddy cultivation.	[64]
4-(2,4-Dichlorophenoxy) Butyrate and 2-(3-Chlorophenoxy) Propionate	Has the dual-guest controlled-release formulation	[65]
2-Methyl-4-Chlorophenoxyacetic	Has sustained release of the AI from the nanopesticides.	[66]
3-glycidyloxypropyl) Trimethoxysilane	The release of the AI was governed by the pseudo-second-order and parabolic-diffusion Models.	[67]
Sodium Dodecyl Sulfate	Improvement of an environmentally friendly pesticide formulation	[68]
Chlorpyrifos/Dodecylbenzenesulfonate	The intercalated pesticide has higher thermal stability. The release rates and equilibrium release amounts of the pesticide were closely dependent on micelles types and release media	[69]

**Table 5:** Nanopesticides prepared using host–guest supramolecular chemistry based on a Zn/Al-LDH host using various guests, AIs, for various targets and advantages.

AI Guest	Target/Advantages	Source(s)
Dazomet	The nanodelivery system also shows better inhibition towards *Ganoderma boninense* growth, to be further explored for combating basal stem rot (BSR) disease in oil palm plantations.	[73]
Quinclorac	To overcome the drawbacks of the overuse of herbicides in paddy cultivation areas	[64]
Mandelic Acid	To be used as a slow-release preservative, and additionally for crop protection	[70]
2,4-Dichlorophenoxy Acetate and 4-Chlorophenoxy Acetate	As a plant growth regulator, controlled release of more than one AI at different controlled rates	[71]
2,4-Dichlorophenoxyacetic Acids @ Glyphosate	Has slow-release properties in decarbonated distilled water, the potential applicability of LDHs as supports for the slow release of acid herbicides	[72]
Quinclorac	Better thermal stability compared to pure herbicide.	[74]
Carbonate Anion	Can be used as an antimicrobial agent	[73]
Hexazonazole	Dual-modal fungicide nanodelivery system, as a fungicide delivery Agent and a micronutrient supplier, to support early plant growth and has the potential to avoid direct contact of fungicides with users due to the intercalation process.	[75]
Nalidixic Acid	Increases the effectiveness for pest control and minimizes possible soil and water contamination	[76]
Nalidixic Acid	Can be used to control pests, infestation and plant disease.	[77]
Pipemidic and Nalidixic Acid	Organic biocides can eliminate almost completely bacteria within a short time	[78]
2-(2,4-Dichlorophenoxy) Acetic Acid, 2-Methyl-4-Chlorophenoxyacetic Acid (MCPA) and 3,5-Dibromo-4-Hydroxybenzonitrile	The Zn/Al-LDH nanoherbicides were prepared by a modified co-precipitation method. The nanoherbicides formed were found to reduce the volatilization by 3-fold and retard leaching through the soil, simultaneously	[79]
Avermectin	Well-control the release of avermectin, a promising candidate for water-dispersible controlled-release formulation	[80]
Bacteriocin Avicin	Potent antimicrobial activity against *Lactobacillus* in a short period (24 h), better production and stability	[81]
Benzoate and its Derivatives	Having a diverse rate of killing depending on the derivatives, the active was released very slowly	[82]
Carboxymethyl Cellulose	Offers the solution for the downside effect of the excessive usage of herbicide in paddy cultivation	[83]
Chitosan	Enhanced antimicrobial effect	[84]
Chlorpyrifos/cyclodextrin	The nanopesticides showed distinct slow release due to the different arrangements of cyclodextrin, the release rate of the AI from nanohybrids was faster and the amount released was higher at pH 6.8 than at pH 5.0	[85]
Pectin/cinnamate acid	Dual applications; cosmetics and crop protection	[86]
Cinnamate anion	Green protective coatings for crops’ protection.	[87]
Ciprofloxacin, sulfanilamide, and oxazolidinone	Offer a promising antimicrobial nanomaterial for various applications	[88]
β-Cypermethrin in sulfonated hydroxyethyl-β-cyclodextrin and sulfobutyl ether β-cyclodextrin	The released amount of AI can be tuned by the pH. Can be used for nanopesticide controlled-release formulation	[89]
Eucalyptus oil	Very effective at very low concentrations, able to kill *Staphylococcus* sp. and inhibit *Pseudomonas aeruginosa* growth	[89]

**Table 6:** Nanopesticides prepared using host–guest supramolecular chemistry based on a Mg/Al-LDH host using various guests, AIs, for various targets and advantages.

AI Guests	Target/Advantages	Source(s)
Atrazine	The herbicide would be delivered close to its site of uptake, enhancing efficiency and reducing the required dose	[96]
4-Chloro-2-Methylphenoxy Acetic Acid	Slow release of the herbicide reduces the hazardous effects that it can pose to the environment.	[93]
2,4-Dichlorophenoxyacetic Acid	A stronger and irreversible herbicidal effect on the test plants	[94]
2-Methyl-4-Chlorophenoxyacetic Acid	Higher loading of the AI herbicide and exhibits better adsorption properties	[95]
Camphorsulfonic Acid and Ciprofloxacin	Remarkable improvement in thermal stability	[97]
Cinnamic Acid	A green pesticide/plant growth regulator	[98]
Hexaconazole and Triadimenol	Loading amount of hexaconazole is higher than triadimenol in the LDHs nanohybrids	[99]
Imazamox	Decreasing water-pollution risk, maintaining efficacy with the advantages of soil compatibility.	[100]
Long-Chain (C-18) Unsaturated Fatty Acid Anions; Elaidate, Oleate, and Linoleate	Reduced affinity of the organo-LDHs to all pesticides, presumably because they lead to structures with reduced hydrophobicity compared to those resulting from the incorporation of linear organic anions.	[101]
Sulfamerazine and Salicylaldehyde	Offer good antimicrobial activity	[102]

**Table 7:** Nanopesticides prepared using host–guest supramolecular chemistry based on various LDH hosts using various guests, AIs, for various targets and advantages.

Hosts	Guests	Target/Advantages	Source(s)
Mg/Fe-LDH and Mg/Al/Fe-LDH	4-Chloro-2-Methylphenoxyacetic acid	Slow release of the herbicide reduces the hazardous effects that it can pose to the environment.	[93]
Co/Cr-LDH	Haloxyfop (anionic form) and insecticide (hexaflumuro, neutral form)	A general and reliable alternative for the analysis of acidic pesticides in anionic and neutral forms.	[108]
Zn/Cu-LDH	Biocides (zinc pyrithione and copper pyrithione)	The nanopesticides demonstrated lower toxicity compared to their free forms, good candidates for antifouling application	[109]
Zn/Cu-LDH	Isomers of hydroxycinnamate	The orthoisomer showed a more sustained release compared to the other isomers	[110]
Li/Al-LDH	4-Chlorophenoxyacetate, 2,4-dichlorophenoxyacetic and 2,4,5-trichlorophenoxyacetate	The intercalation and deintercalation of the AIs were found to be dependent on the type of chlorophenoxyacetic acid used.	[111]

## Data Availability

All data is available in the main text.
